# Chemical Species, Micromorphology, and XRD Fingerprint Analysis of Tibetan Medicine* Zuotai* Containing Mercury

**DOI:** 10.1155/2016/7010519

**Published:** 2016-09-21

**Authors:** Cen Li, Hongxia Yang, Yuzhi Du, Yuancan Xiao, Zhandui, Sanglao, Zhang Wang, Duojie Ladan, Hongtao Bi, Lixin Wei

**Affiliations:** ^1^Pharmacology and Safety Evaluation Key Laboratory of Tibetan Medicine in Qinghai Province, Northwest Institute of Plateau Biology, Chinese Academy of Sciences, Xining, Qinghai 810008, China; ^2^Key Laboratory of Tibetan Medicine Research, Chinese Academy of Sciences, Xining, Qinghai 810008, China; ^3^Tibetan Traditional Medical College, Lhasa, Tibet 850000, China; ^4^Gansu Province Academy of Tibetan Medicine, Hezuo, Gansu 747000, China; ^5^Aba Prefecture Tibetan Medicine Hospital, Maerkang, Sichuan 624000, China; ^6^Qinghai Province Tibetan Medicine Hospital, Xining, Qinghai 810007, China

## Abstract

*Zuotai *(*gTso thal*) is one of the famous drugs containing mercury in Tibetan medicine. However, little is known about the chemical substance basis of its pharmacodynamics and the intrinsic link of different samples sources so far. Given this, energy dispersive spectrometry of X-ray (EDX), scanning electron microscopy (SEM), atomic force microscopy (AFM), and powder X-ray diffraction (XRD) were used to assay the elements, micromorphology, and phase composition of nine* Zuotai *samples from different regions, respectively; the XRD fingerprint features of* Zuotai* were analyzed by multivariate statistical analysis. EDX result shows that* Zuotai* contains Hg, S, O, Fe, Al, Cu, and other elements. SEM and AFM observations suggest that* Zuotai *is a kind of ancient nanodrug. Its particles are mainly in the range of 100–800 nm, which commonly further aggregate into 1–30 *μ*m loosely amorphous particles. XRD test shows that *β*-HgS, S_8_, and *α*-HgS are its main phase compositions. XRD fingerprint analysis indicates that the similarity degrees of nine samples are very high, and the results of multivariate statistical analysis are broadly consistent with sample sources. The present research has revealed the physicochemical characteristics of* Zuotai*, and it would play a positive role in interpreting this mysterious Tibetan drug.

## 1. Introduction

Tibetan medicine is one of the world's existing traditional medicines having completely medical theory, with more than 2000 years' phylogeny [1–3].* Zuotai *(*gTso tha*l), known as “*King of Nectar's Essence*,” is one of the most famous and important drugs in traditional Tibetan medicine [4–7]. It is a kind of black-blue powder with inimitable curative effect and is obtained from liquid silver (mercury), sulfur,* Nengchi Eight Metals*,* Nengchi Eight Minerals*, and other natural drugs through special processing technology by senior Tibetan medicine practitioners [4–6, 8–10].* Zuotai* generally is not used alone, but it is usually used as an adjuvant with other compound drugs [6, 11]. And the doctors of traditional Tibetan medicine have been thinking that* Zuotai* could enhance the efficacy and reduce the toxicity of other drugs [6, 11].* Zuotai* has already been used as a key component of* Renqing* series drugs in Tibetan medicine for 1300 years [12]. By now, it is still used to prepare “*Renqing Mangjue*,” “*Renqing Changjue*,” “*Rannasangpei*,” “*Qishiwei Songshi Wan*,” “*Shanhu Qishiwei Wan*,” “*Zuota Demazi*,” “*Zuozhu Daxi*,” “*Dangzuo,*” and other preparations [5, 11, 12]. These formulations are mainly applied to treat stroke, paralysis, hypertension, neurological disorders, cardiovascular disorders, liver and gallbladder disease, impotence, gastrointestinal diseases, tumors, and so on [5, 6, 8].

As known, heavy metals could pose potentially serious hazard to human health. So, the biological safety and efficacy of* Zuotai* containing mercury need urgently to be reevaluated from a modern medical science perspective. Surprisingly, its toxicity tests suggest that* Zuotai* has no apparently adverse effects on animal subjects under clinical equivalent dose and clinical medication cycle [11, 13–16]. Besides, pharmacological experiments show that* Zuotai* not only can tranquilize and allay excitement, promote sleep, and produce antipyretic effect [17, 18], but also can enhance immunity, have anti-inflammatory effects, extend the life of fruit flies, and inhibit the expression of caspase-3, along with other effects [18–21]. It is confusing why this Tibetan drug containing mercury shows tiny/nontoxicity and various efficacies in animal tests. Biological effects of heavy metals not only relate to doses, but also have closer relationship with their chemical forms; for example, Cr^+3^ is an essential element for mammals with a role in maintaining proper carbohydrate and lipid metabolism; on the contrary, Cr^+6^ has carcinogenic toxicity and genotoxicity [22–25]. So, it is crucial to reveal the chemical species of heavy metals in* Zuotai* for elucidating its pharmacological functions and mechanisms.

Until now, only a few literatures about physicochemical characterization of* Zuotai* are available. Yan [26, 27], Lan et al. [28], and Zhao et al. [29] reported that* Zuotai* was mainly an inorganic mixture of HgS, sulfur, graphite, and other tiny organic molecules, and it was composed of nanoparticles, which further aggregated into microsized particles. However, there still lacks more information about the chemical structure, micromorphology, and XRD fingerprint features of* Zuotai* from large sample sizes. Therefore, in the present research, nine* Zuotai* samples were collected from four major Tibetan regions (Tibet, Qinghai, Gansu, and Sichuan) in China. The element compositions, phase structures, and micromorphology of these samples are analyzed by energy dispersive spectrometry of X-ray (EDX), powder X-ray diffraction (XRD), scanning electron microscopy (SEM), and atomic force microscopy (AFM), respectively. Additionally, the powder XRD fingerprint features of* Zuotai* were analyzed by applying multivariate statistical analysis methods (similarity calculation, systemic cluster analysis, and principal component analysis).

## 2. Materials and Methods

### 2.1. Materials

Nine* Zuotai* samples were kindly provided from the four Tibetan medicine organizations of four major Tibetan regions in China, respectively. The sources of* Zuotai* samples were listed in Table 1. The main technology that processes these samples was well recorded and protected by the patent (number 88107006.8) in China [8]. Before characterization, these samples were sealed in glass bottles and covered by blue cloths according to their traditional storage method.

### 2.2. Measurement of Physicochemical Characteristics

The elements in nine* Zuotai* samples were detected by energy dispersive spectrometry of X-ray (EDX, INCA, Oxford Instruments, Inc.). EDX is a kind of semiquantitative elemental analysis method. The working conditions of EDX are operating voltage of 20 kV, beam spot size of 55 nm, working distance of 8 mm, and hit rates of 2500 cps.

The phase compositions of samples were characterized by powder X-ray diffraction (XRD, X'Pert Pro, PANalytical Company). The working conditions of XRD are Cu target, wavelength of 0.15406 nm, K (*α*1) energy of 40 keV, current intensity of 30 mA, scanning speed of 2°/min, and 2*θ* angle scan range of 5°–80°. Powder X-ray diffraction data were acquired by X'Pert Data Collector and analyzed by X'Pert HighScore Plus using PDF2-2004 of the International Centre for Diffraction Data. Powder XRD spectra were drawn by Origin 8.0. The crystal structure graphs of phase compositions in* Zuotai* were acquired by Diamond 3.2 and FindIt 2009.

The micromorphologies of* Zuotai* were measured by scanning electron microscopy (SEM, JSM-5610LV, Japanese Electronics Co., Ltd.) and atomic force microscopy (AFM, Bioscope Resolve, Bruker Corporation). The working conditions of SEM are LV mode, acceleration voltage of 30 kV, working distance of 8 mm, and working pressure of 1 MPa in electron gun chamber. The working conditions of AFM are ScanAsyst Auto Control, RTESPA-300 probe, scan size of 1 *μ*m, scan rate of 1 Hz, sample/line of 256, peak force amplitude of 150 nm, and* Z* range of 17.9 *μ*m.

### 2.3. Analysis of Powder XRD Fingerprint

The powder XRD fingerprint features were studied with multivariate statistical analysis methods (similarity calculation, systemic cluster analysis, and principal component analysis) by SPSS 22.0 statistical analysis software.

## 3. Results 

### 3.1. Elemental Compositions of* Zuotai*

A large amount of mercury (Hg), sulfur (S), and oxygen (O) was found in nine* Zuotai* samples by EDX analysis. Besides, a small amount of other elements has also been found, such as ferrum (Fe), aluminum (Al), and cuprum (Cu). The results are shown in Table 2 and Supplementary Figure  1 (in Supplementary Material available online at http://dx.doi.org/10.1155/2016/7010519). However, we should note that the oxygen (O) in* Zuotai* may be interfered with inevitably to a certain extent by the residual oxygen from air in the sample testing chamber of EDX. So, whether* Zuotai* samples contain such high level oxygen element needs further study.

### 3.2. Micromorphology of* Zuotai*

**Figure 1:**
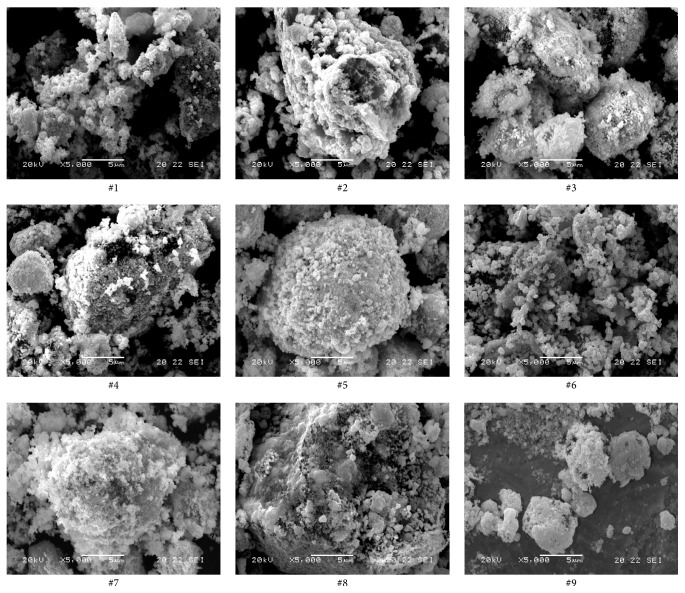
Micron size morphologies of nine Zuotai samples by scanning electron microscopy (SEM). Note: #1 is the sample of Aba Prefecture Tibetan Medicine Hospital; #2 is the sample of Gan'na Prefecture Tibetan Medicine Hospital; #3, #4, and #5 are the samples of the Company of Tibetan Medicine of Tibetan Traditional Medical College; #6, #7, and #8 are the samples of Qinghai Province Tibetan Medicine Hospital; #9 is the sample of the Company of Tibetan Medicine of Tibetan Autonomous Region.

**Figure 2:**
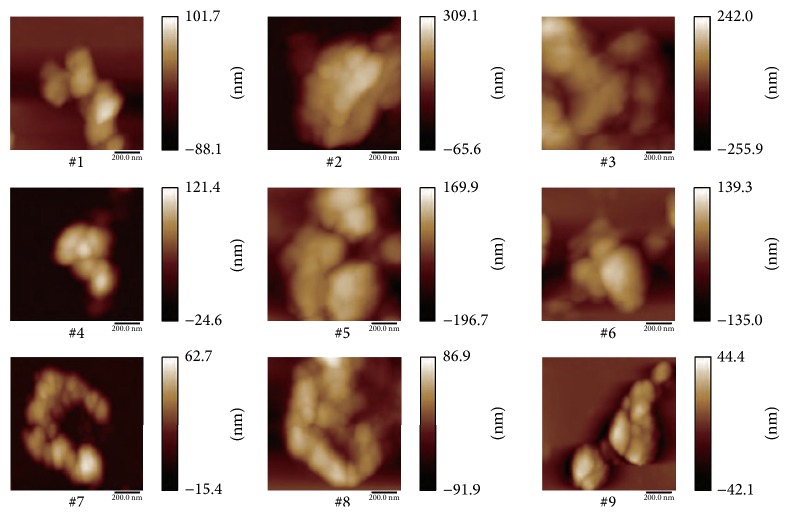
Nanometer size morphologies of nine* Zuotai* samples by atomic force microscopy (AFM). Note: #1 is the sample of Aba Prefecture Tibetan Medicine Hospital; #2 is the sample of Gan'na Prefecture Tibetan Medicine Hospital; #3, #4, and #5 are the samples of the Company of Tibetan Medicine of Tibetan Traditional Medical College; #6, #7, and #8 are the samples of Qinghai Province Tibetan Medicine Hospital; #9 is the sample of the Company of Tibetan Medicine of Tibetan Autonomous Region.

Nine* Zuotai* samples display a variety of micromorphologies, which are composed of a large number of irregular nano-microparticles by scanning electron microscopy (SEM) and atomic force microscopy (AFM), as shown in Figures 1 and 2, respectively. The diameters of these particles vary mostly in the range of 100–800 nm, some even less than 100 nm. And these nano-microparticles commonly further aggregate into 1–30 *μ*m loosely amorphous particles. There are a number of literature reports in both the basic science and pharmaceutics which scientifically define dimensions of nanoparticles ranging in size from 1 to 1000 nm [30–34]. According to this,* Zuotai* should be a kind of ancient nanodrug in traditional Tibetan medicine.

### 3.3. Phase Compositions of* Zuotai*

**Figure 3:**
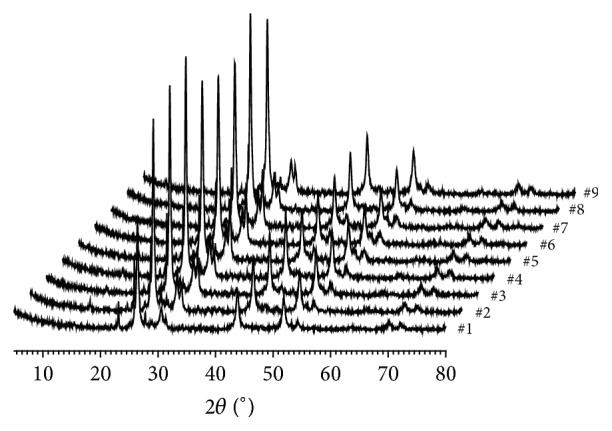
Powder XRD spectrum of nine* Zuotai* samples. Note: #1 is the sample of Aba Prefecture Tibetan Medicine Hospital; #2 is the sample of Gan'na Prefecture Tibetan Medicine Hospital; #3, #4, and #5 are the samples of the Company of Tibetan Medicine of Tibetan Traditional Medical College; #6, #7, and #8 are the samples of Qinghai Province Tibetan Medicine Hospital; #9 is the sample of the Company of Tibetan Medicine of Tibetan Autonomous Region.

**Figure 4:**
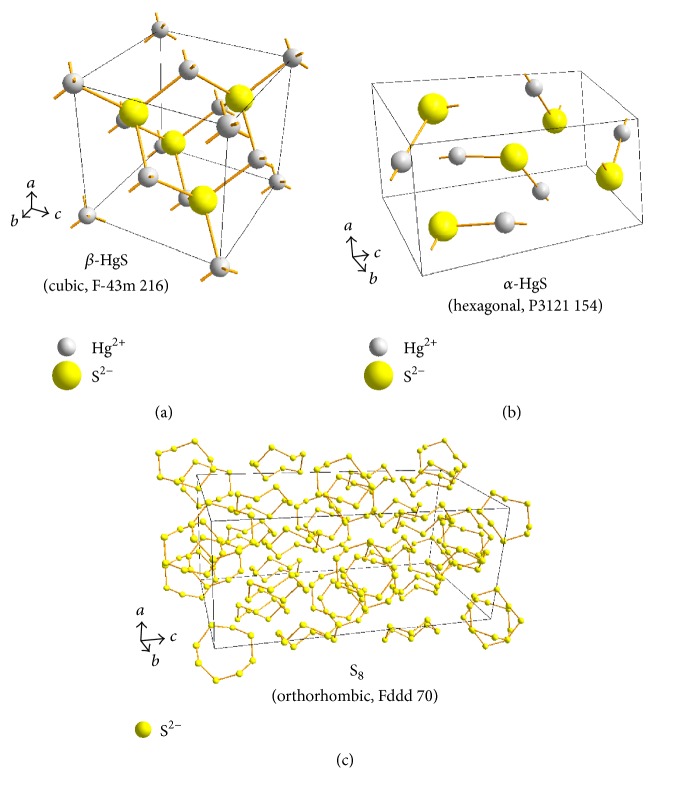
Structures of phase compositions in* Zuotai* samples. Note: (a), (b), and (c) are the crystal structures of *β*-HgS, *α*-HgS, and S_8_, respectively.

Powder XRD analysis found that the common major phase compositions are cubic crystal mercuric sulfide (*β*-HgS) and orthorhombic crystal elemental sulfur (S_8_) in nine* Zuotai* samples (Figure 3, Table 3, and Figure 4). The spacing group of *β*-HgS in* Zuotai* is F-43m (216) with cell parameters *a* = *b* = *c* = 5.8580 and *α* = *β* = *γ* = 90°. The spacing group of S_8_ in nine samples is Fddd (70): *a* = 10.4646, *b* = 12.8660, *c* = 24.4860, and *α* = *β* = *γ* = 90°. Additionally, except for #9 sample, all the other samples contain a small amount of hexagonal crystal mercuric sulfide (*α*-HgS) (Figures 3 and 4 and Table 3). The spacing group of *α*-HgS in the samples from #1 to #8 is P3221 (154) with cell parameters *a* = *b* = 4.1495, *c* = 9.4970, and *α* = *β* = 90°,   *γ* = 120°. Besides, there are also many unknown weak diffraction signals in the nine samples.

This result indicates that the chemical species of mercury in* Zuotai* are cubic crystal mercuric sulfide (*β*-HgS) and hexagonal mercuric sulfide (*α*-HgS) without elemental mercury. Both mercuric sulfides are typical covalent insoluble sulfides. The solubility product constant of *β*-HgS is 1.6 × 10^−52^; the solubility product constant of *α*-HgS is 4.0 × 10^−53^ [35]. So far, there is no definitive evidence showing that the two chemical forms of mercury are toxic in the human body [36, 37].

### 3.4. Powder XRD Fingerprint Analysis of* Zuotai*

#### 3.4.1. Establishing XRD Fingerprint

**Figure 5:**
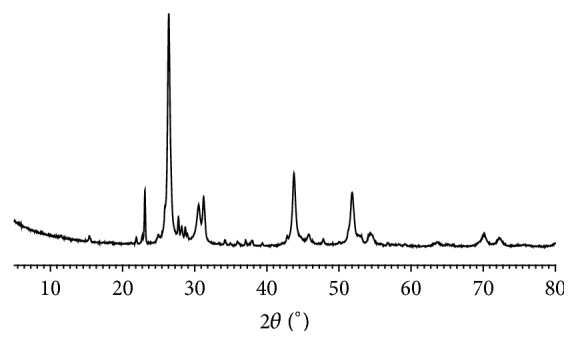
XRD fingerprint of* Zuotai* (average).

**Figure 6:**
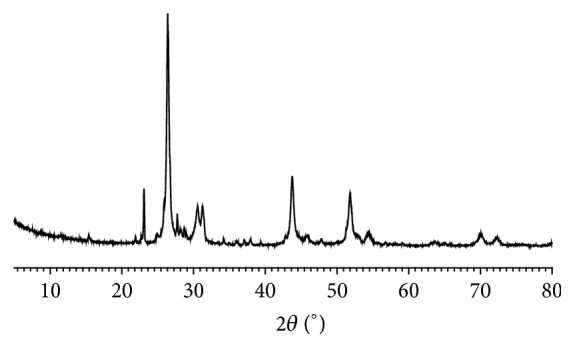
XRD fingerprint of* Zuotai* (median).

The means of diffraction intensity at different 2*θ* angles were acquired by overlaying the diffraction topology spectra of nine samples firstly and then averaging. According to these means, the powder XRD fingerprint (average) of* Zuotai* was drawn by Origin 8.0 (Figure 5). Besides, according to the medians of diffraction intensity of nine samples at different 2*θ* angles, the powder XRD fingerprint (median) was also drawn by Origin 8.0 (Figure 6).

#### 3.4.2. Similarity Calculation of Nine* Zuotai* Samples

The XRD fingerprints (average and median) of* Zuotai* were considered as controls; the similarity degrees (cosine and correlation coefficient) of nine* Zuotai* samples were calculated with SPSS 22.0. The results show that the similarity degrees of the nine samples are very high (all cosines and correlation coefficients > 0.9950) in Table 4.

#### 3.4.3. Cluster Analysis of Nine* Zuotai* Samples

**Figure 7:**
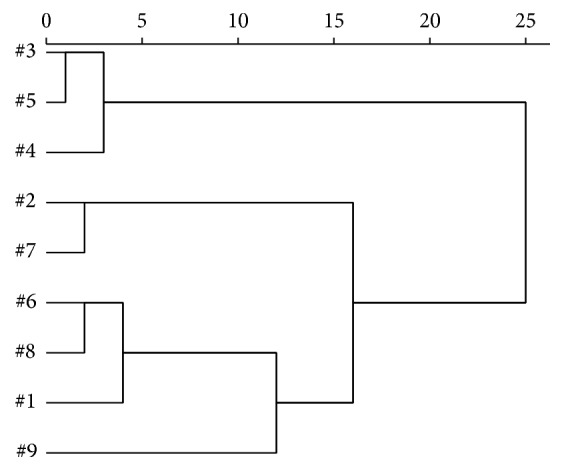
The cluster analysis result of nine* Zuotai* samples. Note: #1 is the sample of Aba Prefecture Tibetan Medicine Hospital; #2 is the sample of Gan'na Prefecture Tibetan Medicine Hospital; #3, #4, and #5 are the samples of the Company of Tibetan Medicine of Tibetan Traditional Medical College; #6, #7, and #8 are the samples of Qinghai Province Tibetan Medicine Hospital; #9 is the sample of the Company of Tibetan Medicine of Tibetan Autonomous Region.

25 common peaks (Supplementary Table 1) are found through analyzing all the diffraction peaks of nine samples in Jade 5.0. And the relative intensities (*I/I*_0_) of 25 common characteristic peaks were considered as indicators; nine* Zuotai* samples were clustered with systematic cluster analysis [38–40] by SPSS 22.0. According to the characteristics of X-ray diffraction analysis data, the between-groups linkage method (*D*_*kl*_ = (1/*n*_*k*_ *n*_*l*_)∑_*x*_*i*_∈*G*_*k*__∑_*x*_*i*_∈*G*_*l*__ *d*_*ij*_) is used to measure the distance between both groups; the Mahalanobis distance method (*d*_*ij*_ = (*x*_*i*_ − *x*_*j*_)′*S* ^−1^(*x*_*i*_ − *x*_*j*_)) is applied to measure the distance between samples, in which *n* refers to sample number, *n*_*k*_ and *n*_*l*_ refer to the *k*th sample and the *l*th sample, *G*_*k*_ and *G*_*l*_ refer to the *k*th group and the *l*th group, *D*_*kl*_ refers to the distance between group *G*_*k*_ and group *G*_*l*_, *d*_*ij*_ refers to the distance between sample *x*_*i*_ and sample *x*_*j*_, and *S* ^−1^ refers to the inverse matrix of sample covariance matrix. The result of cluster analysis is shown in Figure 7.

When the relative distance is 10, nine samples are clustered in four groups: #3, #4, and #5 samples clustering together, #1, #6, and #8 samples clustering together, #2 and #7 samples clustering together, and #9 sample as a group alone. When relative distance is 23, nine samples are clustered in two groups: #1, #2, #3, #4, #5, #6, #7, and #8 samples clustering together and #9 sample still as a single group. Only when relative distance is 25 can all of the samples be clustered together. This result is consistent with the phase composition differences of nine* Zuotai* samples. The samples from #1 to #8 contain cubic mercuric sulfide (*β*-HgS), orthorhombic elemental sulfur (S_8_), and hexagonal mercuric sulfide (*α*-HgS). But #9 sample only contains cubic mercuric sulfide and orthorhombic sulfur, but not hexagonal mercuric sulfide.

#### 3.4.4. Principal Component Analysis (PCA)

Principal component analysis (PCA) is a kind of reducing dimensions method, in that data are projected from high-dimensional space onto low-dimensional space, namely, using a smaller number of new variables instead of a huge number of original variables with minimal information loss [38–41]. In the process of PCA, the absolute intensity of X-ray diffraction can be used. The absolute intensities (*I*) of 25 common peaks of nine* Zuotai* samples were analyzed with PCA by SPSS 22.0, and then the initial eigenvalues (Table 5) and load matrix (Supplementary Table 2) of principal components are acquired.

In Table 5, the initial eigenvalues of the first three principal components are all greater than 2, and the cumulative contribution rate of variance is 83.35 (>80%). Therefore, the first three components (denoted as* F*1,* F*2, and* F*3, resp.) can well explain the inherent information of the original 25 characteristic peaks of nine* Zuotai* samples.

And the variables coefficients of principal component linear equation, namely, standardized eigenvector, can be calculated from the load values of principal components. The standardized eigenvector equation of principal components is ${\hat{t}}_{ij}^{*}=r_{ij}/\sqrt{{\hat{\lambda }}_{j}^{*}}$, in which *r*_*ij*_ refers to the load of principal component, ${\hat{\lambda }}_{j}^{*}$ refers to initial eigenvalues, and ${\hat{t}}_{ij}^{*}$ refers to standardized eigenvector (*i* = 1,2,…, *n*; *j* = 1,2,…, *p*;* n* is sample number;* p* is the indicator number of sample). According to this equation, standardized eigenvectors (shown in Supplementary Table 3) of the first three principal components (*F*1,* F*2, and* F*3) are calculated. The linear equations of the first three principal components (*F*1,* F*2, and* F*3) are obtained through multiplying the standardized eigenvectors with the corresponding normalized peak intensity (*ZX*). The equations of* F*1,* F*2, and* F*3 of Zuotai are as follows:

(1) F1=0.1130ZX1+0.2376ZX2+0.2437ZX3+0.2701ZX4+0.2544ZX5+0.1813ZX6+0.2470ZX7+0.1665ZX8+0.0091ZX9+0.2409ZX10+0.1852ZX11+0.2034ZX12+0.0309ZX13+0.2390ZX14+0.2280ZX15+0.1753ZX16+0.0549ZX17+0.2575ZX18+0.1298ZX19+0.2415ZX20+0.1513ZX21+0.1935ZX22+0.1615ZX23+0.2558ZX24+0.2018ZX25,F2=0.3458ZX1−0.1618ZX2−0.0704ZX3−0.0141ZX4+0.1313ZX5+0.1977ZX6+0.1077ZX7+0.1423ZX8−0.3617ZX9−0.1709ZX10+0.2935ZX11+0.2395ZX12+0.2536ZX13−0.1322ZX14+0.0136ZX15−0.2367ZX16+0.1868ZX17+0.0545ZX18−0.2245ZX19+0.0573ZX20−0.2254ZX21−0.2527ZX22+0.2267ZX23−0.0341ZX24−0.2461ZX25,F3=0.1907ZX1−0.0190ZX2−0.0160ZX3+0.0731ZX4−0.0178ZX5+0.2727ZX6+0.0594ZX7+0.3261ZX8+0.2964ZX9+0.0006ZX10+0.0386ZX11−0.1960ZX12+0.2032ZX13−0.1230ZX14+0.1622ZX15+0.3024ZX16+0.3095ZX17−0.1687ZX18−0.3024ZX19−0.2014ZX20+0.3297ZX21−0.0422ZX22−0.3047ZX23−0.1028ZX24−0.0321ZX25.

**Figure 8:**
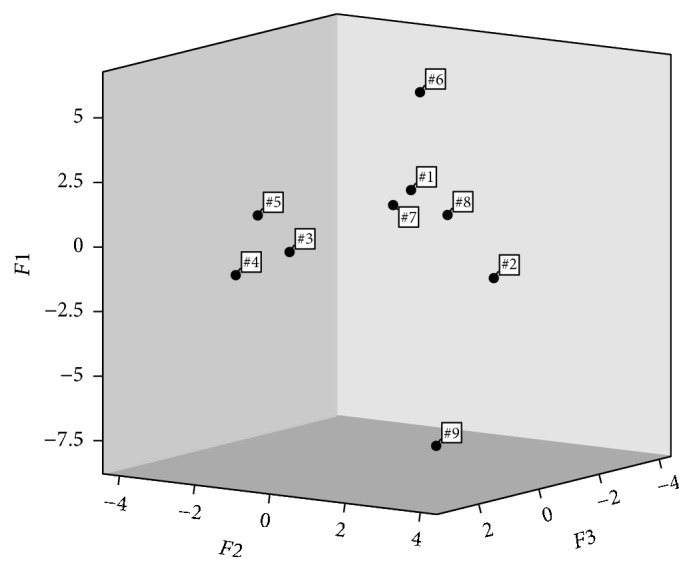
The PCA result of XRD fingerprint of nine* Zuotai* samples. Note: #1 is the sample of Aba Prefecture Tibetan Medicine Hospital; #2 is the sample of Gan'na Prefecture Tibetan Medicine Hospital; #3, #4, and #5 are the samples of the Company of Tibetan Medicine of Tibetan Traditional Medical College; #6, #7, and #8 are the samples of Qinghai Province Tibetan Medicine Hospital; #9 is the sample of the Company of Tibetan Medicine of Tibetan Autonomous Region.

According to the above linear equations of* Zuotai*, the principal component scores of nine* Zuotai* samples were calculated, and the 3D space scatter diagram (Figure 8) of these samples is drawn by SPSS 22.0.

The PCA result shows that nine* Zuotai* samples can be classed into three groups: #1, #2, #6, #7, and #8 are clustered together; #3, #4, and #5 samples are clustered together; #9 sample is clustered as a single group. Moreover, the distance between the first two groups is closer than with the third group on* F*1 coordinate axis. The result of PCA is consistent broadly with systemic cluster analysis.

## 4. Discussion

Tibetan medicine has been playing an important role in the health, reproduction, and development of Tibetan people.* Zuotai* is the key raw material of many precious compound preparations in Tibetan medicine, with tonic effects, reducing toxicity, increasing efficacy, replenishing blood, promoting blood flow, promoting tissue regeneration, invigorating the spleen, and prolonging one's life, among other effects [4–8].* Zuotai* is the common name of “*Renqing Ouqu Zuozhu Qinmu*” in Tibetan medicine. “*Zuo*” means refining, burning, smelting, and cooking; “*Tai*” means ashes, powder, and gray powder; “*Zuotai*” means the powder of burning, burning into powder, and calcining mercury into ash [4, 11, 19, 20, 26, 27, 42–44]. Therefore,* Zuotai* not only is the name of processing product, but also is the name of processing technology [4, 5, 11]. The technology of mercury refining into* Zuotai* was recorded in “*Sibu Yidian*” (*Four Medical Codes*), a Tibetan medicine classic, in the AC 8th century, and was improved by U rgyan pa Rin chen dpal, a famous Tibetan medicine practitioner, in AC 13th century [12, 45]. This technology has had profound influence on the sciences of Tibetan medicine processing, medical formulae, and life cultivation. Almost the alchemy of Chinese Taoist and the mercury praeparatum technology of Indian Tantra had already gone away with history, but only the processing technology of* Zuotai* survives and flourishes till today; this may have been because* Zuotai* has been used as a core adjuvant in many precious Tibetan medicine component preparations [45, 46]. Meanwhile, this conserves a kind of unique ancient technology for traditional Tibetan culture. In 2006, the processing technology of* Zuotai* has been collected in the Chinese Intangible Cultural Heritage List [47].

Nanometer is not only a concept of spatial scale, but also a new way of thinking. The present research found that* Zuotai* particles are mainly in 100–800 nm range, and some are less than 100 nm, which commonly aggregate into about 1–30 *μ*m loosely amorphous particles. This is consistent with the reports of Yan et al. [26], Lan et al. [28], and Zhao et al. [29]. In the pharmaceutical field, the particle size of a nanodrug is generally defined in the range from 1 nm to 1000 nm [30–34]. So, this indicates that* Zuotai* is a kind of ancient nanodrug. There are three main absorption ways of oral nanoparticles in the intestinal tract [48, 49]: (1) paracellular passage—particles “kneading” between intestinal epithelial cells due to their extremely small size (<50 nm); (2) endocytotic uptake—particles absorbed by intestinal enterocytes through endocytosis (particles size < 500 nm); (3) lymphatic uptake—particles adsorbed by M cells of Peyer's patches (particle size < 5 *μ*m). It is surprising to find that the scale of* Zuotai* particles coincides with modern nanodrug concept. This finding greatly arouses our interests in this ancient nanodrug. According to the above absorption ways of nanodrugs in the intestine,* Zuotai* particles may mainly be absorbed through the intestinal epithelial cells and the M cells of Peyer's patches.

Accurate characterization for the chemical species of heavy metals in* Zuotai* is the prerequisite of understanding its peculiarly biological effects. The present study suggests that* Zuotai* contains cubic *α*-HgS (F-43m, 216), orthorhombic S_8_ (Fddd, 70), and hexagonal *α*-HgS (P3221, 154). Besides, there are still many unknown very weak X-ray diffraction signals, which are difficult to analyze. According to the processing technology of* Zuotai*, we can know that it is obtained through mixing the burned* Nengchi Eight Eshes* and the burned* Nengchi Eight Mineral Ashes* into the prepared mercury powder at 3.3% rate, respectively. The chemical components of* Nengchi Eight Eshes* and* Nengchi Eight Mineral Ashes* have been reported [50, 51] by some authors of the present paper. The other phase compositions of* Zuotai* can be deduced from the* Nengchi Eight Eshes* and the refined* Nengchi Eight Mineral Ashes*. Therefore, except for the main phase compositions above,* Zuotai* also should contain minor or trace quadratic AuPb_2_ (I4/mcm, 144), quadratic PbO (P4/nmm, 129), orthorhombic PbO (Pbma, 57), cubic Pb (Fm-3m, 225), monoclinic Ag_2_S (P21/n, 14), quadratic Cu_1.98_(Zn_0.73_Fe_0.29_)Sn_0.99_S_4_(I-4, 82), hexagonal CuS (P63/mmc, 194), hexagonal SiO_2_ (P3121, 152), hexagonal NaCu_2_S_2_ (P3m1, 156), rhomboidal Ca(Fe_+2_,Mg)(CO_3_)_2_(R-3, 148), orthorhombic Cu_7_S_4_ (Pnma, 62), monoclinic Cu_7_S_4_ (C2/m, 12), monoclinic CuO (C2/c, 15), hexagonal FeS (P-62c, 192), cubic PbS (Fm-3m, 226), cubic ZnS (F-43m, 216), rhomboidal CaCO_3_ (R-3c, 167), cubic Fe_+2_Fe_2+3_O_4_ (namely, Fe_3_O_4_; Fd-3m, 227), orthorhombic SnS (Pnma, 62), orthorhombic PbSO_4_ (Pnma, 62), hexagonal SnS_2_ (P63mc, 186), cubic KCl (Fm-3m, 225), monoclinic K(Mg,Fe)_3_(Al,Fe)Si_3_O_10_(OH,F)_2_ (C2/m, 12), orthorhombic Mg_2_SiO_4_ (Pbnm, 62), monoclinic KMg_3_Si_3_AlO_10_(F,OH)_2_ (C2/m, 12), triclinic (Na_0.4_Ca_0.6_)Al_1.6_Si_2.4_O_8_ (unknown spacing group), orthorhombic FeAs (Pnma, 62), monoclinic K_2_Ca(SO_4_)_2_·H_2_O (P21/m, 11), orthorhombic FeAs_2_ (Pnnm, 58), quadratic Fe_2_As (P4/nmm, 129), rhombic-hexahedral CaCO_3_ (R-3c, 167), cubic Cu_2_S (Fm-3m, 225), and so on [45, 47]. Besides, Yan et al. [26, 27] and Lan et al. [28] reported that* Zuotai* may also contain some amount of FeC, *ε*-Fe_2_O_3_, (S_4_N_3_)Cl, C, and trace organics. As seen from the above, the chemical components and crystal structures of* Zuotai* are extremely complex, and this may be the substance basis of its peculiar curative effects.

In summary, the present research suggests that mercury (Hg) and sulfur (S) are the major elements, and ferrum (Fe), aluminum (Al), and cuprum (Cu) are the minor metal elements in* Zuotai*. The chemical species of mercury in* Zuotai* are insoluble cubic mercuric sulfide (*β*-HgS) and minor hexagonal mercuric sulfide (*α*-HgS). And orthorhombic sulfur (S_8_) is also the main phase composition in* Zuotai*. Moreover,* Zuotai* is a kind of ancient nanodrug in traditional Tibetan medicine. Its particles are mainly in the range from 100 nm to 800 nm and further aggregate into 1–30 *μ*m loosely amorphous particles. Besides, the intrinsic relationship among samples from different sources is revealed through multivariate statistical analysis, according to the established powder XRD fingerprint of* Zuotai*. As an important drug containing heavy metals in Tibetan medicine,* Zuotai* has many mysteries, which are worth exploring. Science always has its historical limitation, and if the knowledge of contemporary science cannot explain it, we cannot abandon it blindly, but it needs to be handed down, waiting for the science of the future to explain it.

## Supplementary Material

This Supplementary Materials are composed by one supplementary figure (EDX Spectrum of Nine Zuotai Samples) and four supplementary tables (Peaks Position (2θ/°) and Relative Intensity (I /I_0_) of 25 Common XRD Peaks in Nine Zuotai Samples, Load Matrix of Principal Components, Standardized Eigenvector Matrix, and The Lattice Distances (Å) of Nine Zuotai Samples XRD Peaks). These materials could provide further experimental data details for this research paper, and could provide more reference for some interested researchers.

## Figures and Tables

**Table 1:** Source of nine *Zuotai* samples.

Number	Sample source	Comments
#1	Aba Prefecture Tibetan Medicine Hospital, Maerkang, Sichuan, China	Collected on November 11, 2010
#2	Gan'na Prefecture Tibetan Medicine Hospital, Hezuo, Gansu, China	Collected on November 2, 2010
#3	The Company of Tibetan Medicine of Tibetan Traditional Medical College, Lhasa, Tibet, China	Processed in August 2006
#4	The Company of Tibetan Medicine of Tibetan Traditional Medical College, Lhasa, Tibet, China	Processed in September 2007
#5	The Company of Tibetan Medicine of Tibetan Traditional Medical College, Lhasa, Tibet, China	Processed in September 2008
#6	Qinghai Province Tibetan Medicine Hospital, Xining, Qinghai, China	Processed in August 2010
#7	Qinghai Province Tibetan Medicine Hospital, Xining, Qinghai, China	Processed in September 2009
#8	Qinghai Province Tibetan Medicine Hospital, Xining, Qinghai, China	Processed in September 2008
#9	The Company of Tibetan Medicine of Tibetan Autonomous Region, Lhasa, Tibet, China	Collected on January 8, 2010

**Table 2:** Elemental contents in nine *Zuotai *samples by energy dispersive spectrometry of X-ray (EDX).

Samples	Items	O	Al	S	Fe	Cu	Hg
#1	Weight %	6.36	0.42	23.06	0.62	0.83	68.72
	Atomic %	26.52	1.04	47.98	0.74	0.87	22.86

#2	Weight %	6.44	—	38.98	0.67	—	53.92
	Atomic %	21.19	—	64.03	0.63	—	14.16

#3	Weight %	4.06	—	32.44	—	—	63.5
	Atomic %	16.03	—	63.96	—	—	20.01

#4	Weight %	3.01	—	31.89	—	—	65.1
	Atomic %	12.5	—	65.97	—	—	21.53

#5	Weight %	3.71	—	31.11	—	—	65.18
	Atomic %	15.18	—	63.54	—	—	21.28

#6	Weight %	4.09	—	20.14	0.53	—	75.24
	Atomic %	20.16	—	49.52	0.75	—	29.58

#7	Weight %	3.36	—	20.8	0.71	—	75.12
	Atomic %	16.87	—	52.06	1.02	—	30.05

#8	Weight %	3.22	—	22.57	0.6	—	73.61
	Atomic %	15.67	—	54.88	0.84	—	28.61

#9	Weight %	2.76	—	22.52	0.66	—	74.06
	Atomic %	13.75	—	55.91	0.94	—	29.39

Note: #1 is the sample of Aba Prefecture Tibetan Medicine Hospital; #2 is the sample of Gan'na Prefecture Tibetan Medicine Hospital; #3, #4, and #5 are the samples of the Company of Tibetan Medicine of Tibetan Traditional Medical College; #6, #7, and #8 are the samples of Qinghai Province Tibetan Medicine Hospital; #9 is the sample of the Company of Tibetan Medicine of Tibetan Autonomous Region.

**Table 3:** Phase compositions in nine *Zuotai *samples by powder XRD.

Samples	ICDD	Compound	Chem. form.	Crystal system	Space group
#1	01-075-1538	Metacinnabar	HgS	Cubic	F-43m (216)
	01-078-1889	Sulfur, alpha	S_8_	Orthorhombic	Fddd (70)
	00-042-1408	Vermilion	HgS	Hexagonal	P3221 (154)

#2	01-075-1538	Metacinnabar	HgS	Cubic	F-43m (216)
	01-078-1889	Sulfur, alpha	S_8_	Orthorhombic	Fddd (70)
	00-042-1408	Vermilion	HgS	Hexagonal	P3221 (154)

#3	01-075-1538	Metacinnabar	HgS	Cubic	F-43m (216)
	01-078-1889	Sulfur, alpha	S_8_	Orthorhombic	Fddd (70)
	00-042-1408	Vermilion	HgS	Hexagonal	P3221 (154)

#4	01-075-1538	Metacinnabar	HgS	Cubic	F-43m (216)
	01-078-1889	Sulfur, alpha	S_8_	Orthorhombic	Fddd (70)
	00-042-1408	Vermilion	HgS	Hexagonal	P3221 (154)

#5	01-075-1538	Metacinnabar	HgS	Cubic	F-43m (216)
	01-078-1889	Sulfur, alpha	S_8_	Orthorhombic	Fddd (70)
	00-042-1408	Vermilion	HgS	Hexagonal	P3221 (154)

#6	01-075-1538	Metacinnabar	HgS	Cubic	F-43m (216)
	01-078-1889	Sulfur, alpha	S_8_	Orthorhombic	Fddd (70)
	00-042-1408	Vermilion	HgS	Hexagonal	P3221 (154)

#7	01-075-1538	Metacinnabar	HgS	Cubic	F-43m (216)
	01-078-1889	Sulfur, alpha	S_8_	Orthorhombic	Fddd (70)
	00-042-1408	Vermilion	HgS	Hexagonal	P3221 (154)

#8	01-075-1538	Metacinnabar	HgS	Cubic	F-43m (216)
	01-078-1889	Sulfur, alpha	S_8_	Orthorhombic	Fddd (70)
	00-042-1408	Vermilion	HgS	Hexagonal	P3221 (154)

#9	01-075-1538	Metacinnabar	HgS	Cubic	F-43m (216)
	01-078-1889	Sulfur, alpha	S_8_	Orthorhombic	Fddd (70)

Note: #1 is the sample of Aba Prefecture Tibetan Medicine Hospital; #2 is the sample of Gan'na Prefecture Tibetan Medicine Hospital; #3, #4, and #5 are the samples of the Company of Tibetan Medicine of Tibetan Traditional Medical College; #6, #7, and #8 are the samples of Qinghai Province Tibetan Medicine Hospital; #9 is the sample of the Company of Tibetan Medicine of Tibetan Autonomous Region.

**Table 4:** The similarity of XRD fingerprints of nine *Zuotai* samples.

Samples	Average		Median	
	Cosine	Correlation	Cosine	Correlation
#1	0.9991	0.9988	0.9989	0.9986
#2	0.9980	0.9980	0.9978	0.9976
#3	0.9988	0.9985	0.9979	0.9974
#4	0.9982	0.9980	0.9975	0.9974
#5	0.9986	0.9983	0.9981	0.9978
#6	0.9989	0.9987	0.9994	0.9993
#7	0.9983	0.9984	0.9980	0.9979
#8	0.9990	0.9986	0.9994	0.9993
#9	0.9974	0.9968	0.9966	0.9958

Note: #1 is the sample of Aba Prefecture Tibetan Medicine Hospital; #2 is the sample of Gan'na Prefecture Tibetan Medicine Hospital; #3, #4, and #5 are the samples of the Company of Tibetan Medicine of Tibetan Traditional Medical College; #6, #7, and #8 are the samples of Qinghai Province Tibetan Medicine Hospital; #9 is the sample of the Company of Tibetan Medicine of Tibetan Autonomous Region.

**Table 5:** Initial eigenvalues of principal components.

Component	Initial eigenvalues		
	Total	% of variance	Cumulative %
1	13.16	52.641	52.641
2	4.843	19.372	72.013
3	2.834	11.337	83.35
4	1.631	6.522	89.872
5	1.074	4.296	94.168
6	0.669	2.677	96.845
7	0.571	2.284	99.128
⋮	⋮	⋮	⋮
25	−6.03*E* − 16	−2.41*E* − 15	100
