# Supplementary material for: Chemical Species, Micromorphology, and XRD Fingerprint Analysis of Tibetan Medicine Zuotai Containing Mercury
Source: Bioinorg Chem Appl. 2016 Sep 21;2016:7010519. doi: 10.1155/2016/7010519 (PMC5050389; doi:10.1155/2016/7010519)
Supplement: Supplementary file 1 — This Supplementary Materials are composed by one supplementary figure (EDX Spectrum of Nine Zuotai Samples) and four supplementary tables (Peaks Position (2θ/°) and Relative Intensity (I /I0) of 25 Common XRD Peaks in Nine Zuotai Samples, Load Matrix of Principal Components, Standardized Eigenvector Matrix, and The Lattice Distances (Å) of Nine Zuotai Samples XRD Peaks). These materials could provide further experimental data details for this research paper, and could provide more reference for some interested researchers. [file 7010519.f1.pdf]

Supplementary Materials Lists

***Supplementary Figure 1*: EDX Spectrum of Nine *Zuotai* Samples**

***Supplementary Table 1* : Peaks Position (2θ/°) and Relative Intensity (I/I_0) of 25 Common XRD Peaks in Nine *Zuotai* Samples**

***Supplementary Table 2*: Load Matrix of Principal Components**

***Supplementary Table 3*: Standardized Eigenvector Matrix**

***Supplementary Table 4*: The Lattice Distances (Å) of Nine *Zuotai* Samples XRD Peaks**

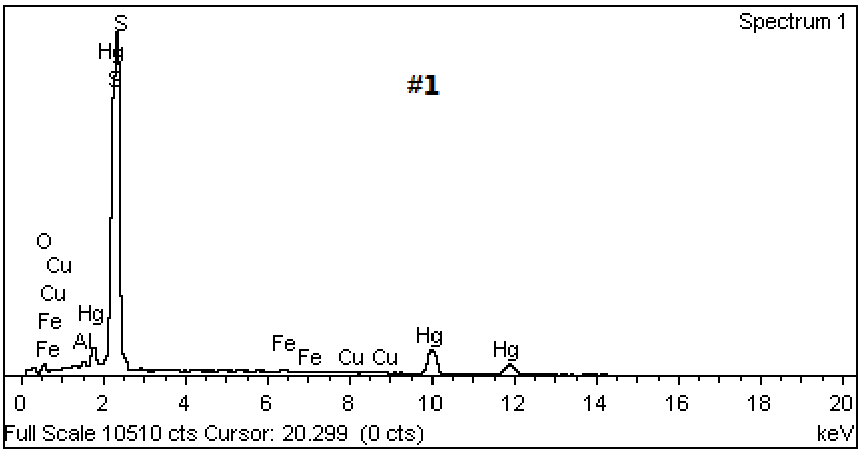
Spectrum 1
#1
S
Hg
S
O
Cu
Cu
Fe
Fe
Hg
Al
Fe
Fe
Cu Cu
Hg
Hg
0 2 4 6 8 10 12 14 16 18 20
Full Scale 10510 cts Cursor: 20.299 (0 cts)
keV

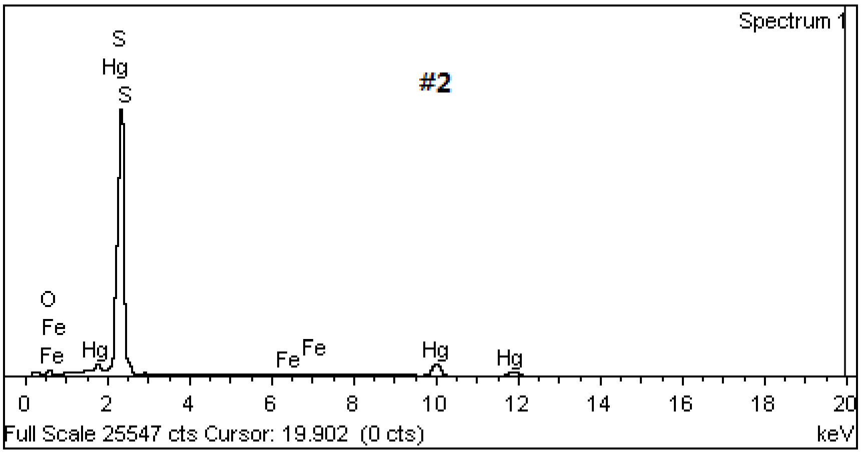
Spectrum 1
#2
S
Hg
S
O
Fe
Fe
Hg
Fe
Fe
Hg
Hg
0 2 4 6 8 10 12 14 16 18 20
Full Scale 25547 cts Cursor: 19.902 (0 cts)
keV

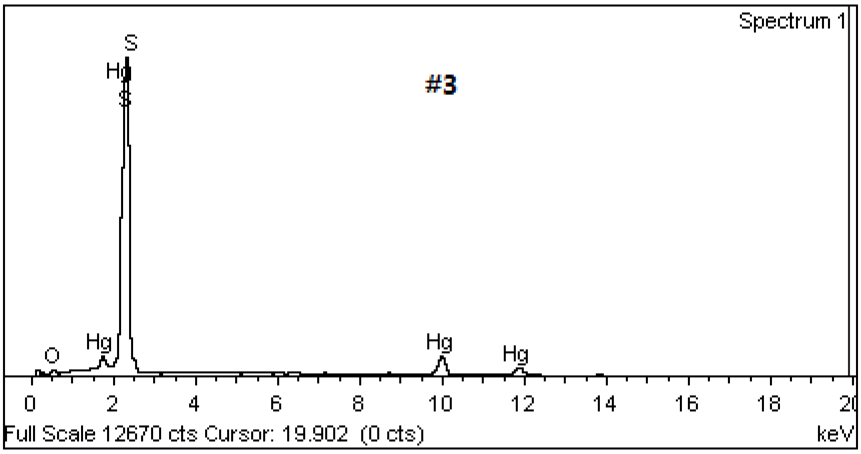
Spectrum 1
#3
S
Hg
S
O
Hg
Hg
Hg
0 2 4 6 8 10 12 14 16 18
Full Scale 12670 cts Cursor: 19.902 (0 cts)
keV

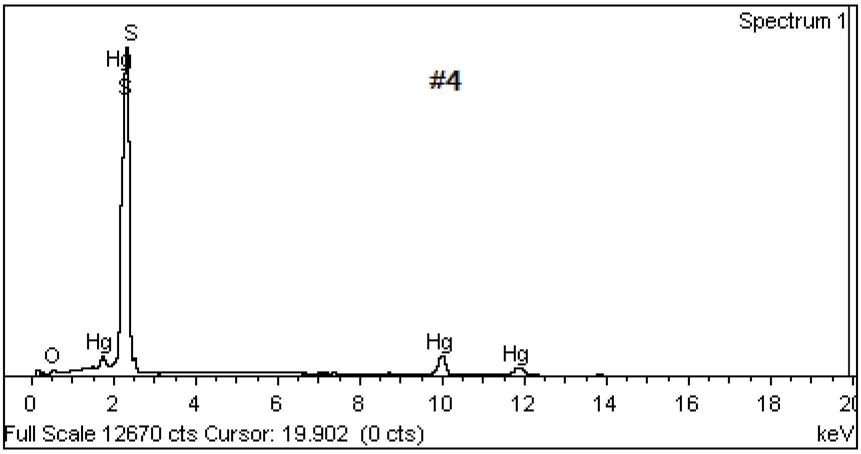
Spectrum 1
#4
S
Hg
S
O
Hg
Hg
Hg
0 2 4 6 8 10 12 14 16 18
Full Scale 12670 cts Cursor: 19.902 (0 cts)
keV

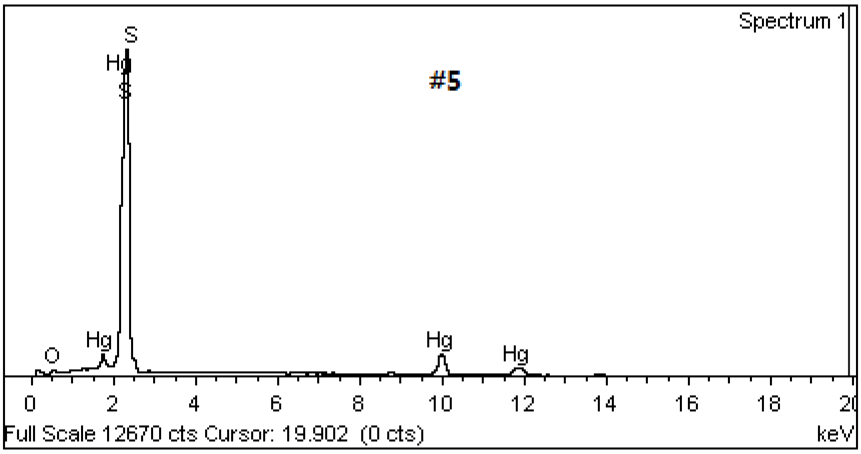
Spectrum 1
#5
S
Hg
S
O
Hg
Hg
Hg
0 2 4 6 8 10 12 14 16 18 20
Full Scale 12670 cts Cursor: 19.902 (0 cts)
keV

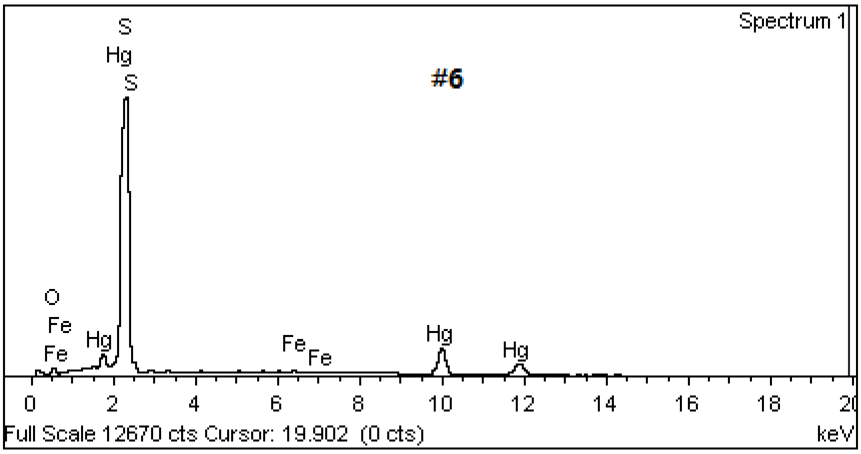
Spectrum 1
#6
S
Hg
S
O
Fe
Fe
Hg
Fe
Fe
Hg
Hg
0 2 4 6 8 10 12 14 16 18 20
Full Scale 12670 cts Cursor: 19.902 (0 cts)
keV

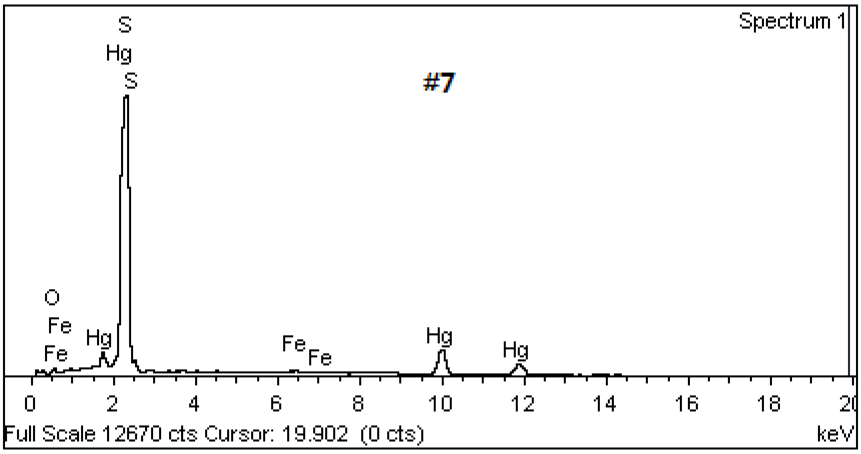
Spectrum 1
#7
S
Hg
S
O
Fe
Fe
Hg
Fe
Fe
Hg
Hg
0 2 4 6 8 10 12 14 16 18 20
Full Scale 12670 cts Cursor: 19.902 (0 cts)
keV

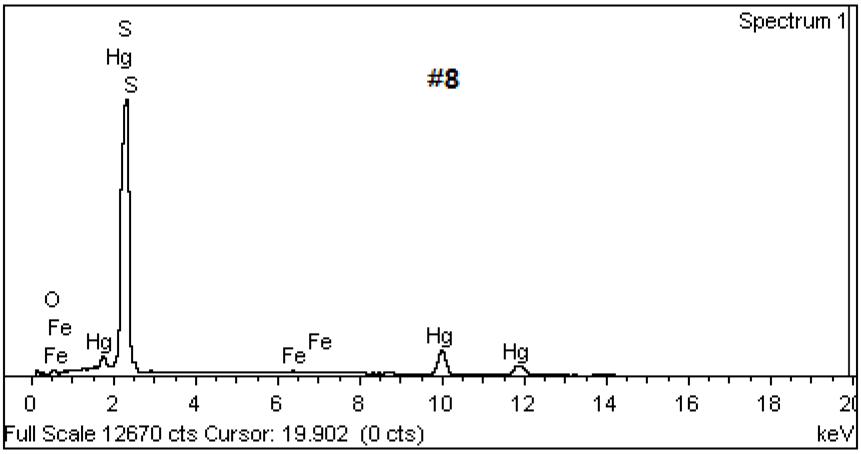
Spectrum 1
#8
S
Hg
S
O
Fe
Fe
Hg
Fe
Fe
Hg
Hg
0 2 4 6 8 10 12 14 16 18 20
Full Scale 12670 cts Cursor: 19.902 (0 cts)
keV

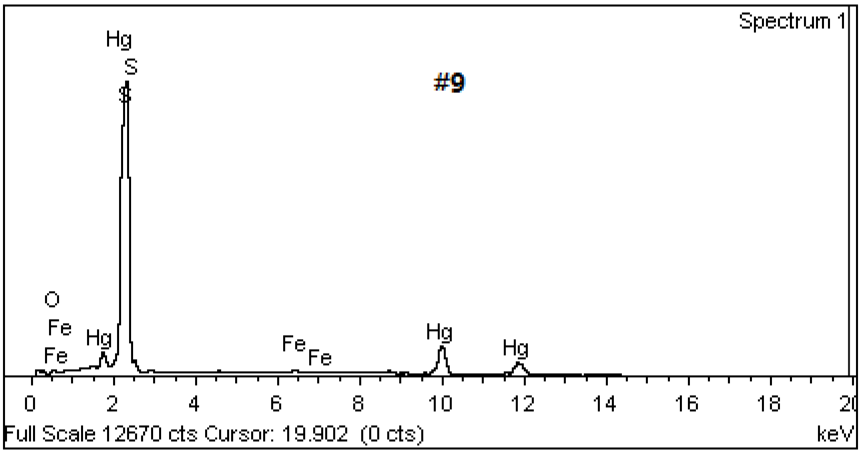

Supplementary Figure 1 **EDX Spectrum of Nine *Zuotai* Samples**

(Note: #1is the sample of Aba Prefecture Tibetan Medicine Hospital; #2 is the sample of Gan'na Prefecture Tibetan Medicine Hospital; #3, #4 and #5 are the samples of the Company of Tibetan Medicine of Tibetan Traditional Medical College; #6, #7 and #8 are the samples of Qinghai Province Tibetan Medicine Hospital; #9 is the sample of the Company of Tibetan Medicine of Tibetan Autonomous Region).

Supplementary Table 1 **Peaks Position (2θ/°) and Relative Intensity (I/I_0) of 25 Common XRD Peaks in Nine *Zuotai* Samples**

Common peaks	Average		RSD		I/I0（%）										
	2θ	d	2θ	d	#1	#2	#3	#4	#5	#6	#7	#8	#9	Average	Median
1	15.42	5.75	0.28%	0.28%	2.57	3.31	2.45	3.01	3.26	2.71	2.39	4.53	4.09	3.15	3.01
2	21.92	4.06	0.20%	0.19%	3.52	2.09	4.18	3.21	3.53	3.30	2.90	3.43	2.58	3.19	3.30
3	22.71	3.92	0.20%	0.20%	3.89	2.48	3.70	3.43	4.24	3.91	2.75	3.63	3.60	3.51	3.63
4	23.11	3.85	0.16%	0.16%	28.20	21.49	27.42	30.86	29.26	29.10	23.28	29.05	24.40	27.01	28.20
5	24.96	3.57	0.16%	0.15%	3.07	2.82	2.69	3.17	3.15	3.46	2.74	3.17	3.28	3.06	3.15
6	25.87	3.44	0.15%	0.15%	14.66	16.18	15.67	18.46	16.14	13.17	12.82	16.84	18.67	15.85	16.14
7	26.39	3.38	0.16%	0.16%	100.00	100.00	100.00	100.00	100.00	100.00	100.00	100.00	100.00	100.00	100.00
8	27.76	3.21	0.15%	0.15%	9.85	9.38	9.69	13.00	10.78	8.85	7.01	12.44	11.66	10.30	9.85
9	28.20	3.16	0.20%	0.20%	3.58	2.54	10.12	9.50	9.74	2.04	5.24	2.44	3.36	5.40	3.58
10	28.70	3.11	0.14%	0.13%	6.54	3.42	5.70	5.93	7.02	5.89	4.59	5.14	4.15	5.38	5.70
11	28.98	3.08	0.16%	0.16%	2.32	2.30	2.02	2.57	2.18	2.50	2.36	3.24	3.08	2.51	2.36
12	30.55	2.93	0.22%	0.22%	16.35	15.79	13.35	13.92	13.14	14.95	15.05	16.10	17.37	15.11	15.05
13	34.19	2.62	0.13%	0.12%	1.69	2.27	2.52	1.94	2.58	1.86	1.71	2.80	3.61	2.33	2.27
14	35.92	2.50	0.12%	0.12%	2.11	1.23	2.36	1.89	1.69	2.19	1.70	1.77	1.45	1.82	1.77
15	37.07	2.41	0.09%	0.09%	2.16	2.15	2.17	3.15	2.85	3.09	1.92	2.23	2.28	2.44	2.23
16	37.93	2.37	0.14%	0.12%	1.79	1.57	2.75	2.81	2.59	1.75	1.78	1.91	1.37	2.04	1.79
17	39.38	2.29	0.08%	0.07%	0.97	1.15	0.94	1.81	1.11	1.10	1.16	1.23	1.87	1.26	1.15
18	43.77	2.07	0.11%	0.10%	33.05	27.84	30.84	30.63	28.84	28.94	26.5	28.59	34.22	29.94	28.94
19	47.80	1.90	0.17%	0.10%	3.38	1.80	2.71	2.21	2.33	1.71	1.98	1.89	2.18	2.24	2.18
20	51.81	1.76	0.10%	0.09%	23.74	19.82	21.69	22.96	20.17	20.92	20.25	20.01	26.43	21.78	20.92
21	54.43	1.69	0.33%	0.31%	4.16	3.90	5.26	5.77	4.85	3.70	3.66	3.84	5.21	4.48	4.16
22	56.76	1.62	0.05%	0.05%	1.60	0.66	1.60	1.31	1.63	1.33	0.97	1.21	1.20	1.28	1.31
23	63.68	1.46	0.08%	0.07%	1.60	1.47	1.23	1.08	1.24	1.28	1.09	1.33	1.68	1.33	1.28
24	70.09	1.34	0.08%	0.07%	4.42	3.82	4.66	4.48	4.27	3.70	3.84	3.98	5.37	4.28	4.27
25	72.24	1.31	0.17%	0.13%	2.99	1.88	3.21	3.23	2.83	2.55	2.68	2.53	3.29	2.80	2.83

(Note: #1 is the sample of Aba Prefecture Tibetan Medicine Hospital; #2 is the sample of Gan'na Prefecture Tibetan Medicine Hospital; #3, #4 and #5 are the samples of the Company of Tibetan Medicine of Tibetan Traditional Medical College; #6, #7 and #8 are the samples of Qinghai Province Tibetan Medicine Hospital; #9 is the sample of the Company of Tibetan Medicine of Tibetan Autonomous Regio

Supplementary Table 2 **Load Matrix of Principal Components**

Peak	Load values			Peak	Load values		
	F1	F2	F3		F1	F2	F3
1	0.410	0.761	0.321	14	0.867	-0.291	-0.207
2	0.862	-0.356	-0.032	15	0.827	0.03	0.273
3	0.884	-0.155	-0.027	16	0.636	-0.521	0.509
4	0.98	-0.031	0.123	17	0.199	0.411	0.521
5	0.923	0.289	-0.030	18	0.934	0.12	-0.284
6	0.658	0.435	0.459	19	0.471	-0.494	-0.509
7	0.896	0.237	-0.100	20	0.876	0.126	-0.339
8	0.604	0.313	0.549	21	0.549	-0.496	0.555
9	0.033	-0.796	0.499	22	0.702	-0.556	-0.071
10	0.874	-0.376	0.001	23	0.586	0.499	-0.513
11	0.672	0.646	0.065	24	0.928	-0.075	-0.173
12	0.738	0.527	-0.330	25	0.732	-0.542	-0.054
13	0.112	0.558	0.342				

Supplementary Table 3 **Standardized Eigenvector Matrix**

Peak	Eigenvector			Peak	Eigenvector		
	F1	F2	F3		F1	F2	F3
1	0.1130	0.3458	0.1907	14	0.2390	-0.1322	-0.1230
2	0.2376	-0.1618	-0.0190	15	0.2280	0.0136	0.1622
3	0.2437	-0.0704	-0.0160	16	0.1753	-0.2367	0.3024
4	0.2701	-0.0141	0.0731	17	0.0549	0.1868	0.3095
5	0.2544	0.1313	-0.0178	18	0.2575	0.0545	-0.1687
6	0.1814	0.1977	0.2727	19	0.1298	-0.2245	-0.3024
7	0.2470	0.1077	-0.0594	20	0.2415	0.0573	-0.2014
8	0.1665	0.1422	0.3261	21	0.1513	-0.2254	0.3297
9	0.0091	-0.3617	0.2964	22	0.1935	-0.2527	-0.0422
10	0.2410	-0.1709	0.0006	23	0.1615	0.2267	-0.3047
11	0.1852	0.2935	0.0386	24	0.2558	-0.0341	-0.1028
12	0.2034	0.2395	-0.1960	25	0.2018	-0.2461	-0.0321
13	0.0309	0.2536	0.2032				

Supplementary Table 4 **The Lattice Distances (Å) of Nine *Zuotai* Samples XRD Peaks**

**Peaks**		**#1**	**#2**	**#3**	**#4**	**#5**	**#6**	**#7**	**#8**	**#9**
1	2θ	8.91	15.38	15.45	15.39	11.50	11.48	11.50	11.42	15.45
	d	9.93	5.76	5.73	5.76	7.69	7.71	7.69	7.75	5.73
2	2θ	11.54	21.88	21.96	21.89	15.42	15.39	15.42	15.37	21.94
	d	7.67	4.06	4.05	4.06	5.74	5.76	5.75	5.76	4.05
3	2θ	15.50	22.64	22.75	22.68	21.91	21.89	21.93	21.86	22.74
	d	5.72	3.93	3.91	3.92	4.06	4.06	4.05	4.07	3.91
4	2θ	22.00	23.07	23.15	23.08	22.72	22.72	22.73	22.66	23.12
	d	4.04	3.86	3.84	3.85	3.91	3.91	3.91	3.92	3.85
5	2θ	22.79	24.93	24.96	24.93	23.10	23.08	23.11	23.06	24.98
	d	3.90	3.57	3.57	3.57	3.85	3.85	3.85	3.86	3.56
6	2θ	23.18	25.82	25.90	25.84	24.97	24.91	24.96	24.94	25.89
	d	3.84	3.45	3.44	3.45	3.57	3.57	3.57	3.57	3.44
7	2θ	25.04	26.34	26.41	26.35	25.87	25.84	25.87	25.82	26.42
	d	3.56	3.38	3.37	3.38	3.44	3.45	3.44	3.45	3.37
8	2θ	25.94	27.70	27.80	27.73	26.40	26.39	26.42	26.35	27.79
	d	3.44	3.22	3.21	3.22	3.38	3.38	3.37	3.38	3.21
9	2θ	26.47	28.16	28.23	28.13	27.75	26.72	27.75	27.71	28.26
	d	3.37	3.17	3.16	3.17	3.21	3.34	3.21	3.22	1.16
10	2θ	27.82	28.67	28.74	28.69	28.20	27.73	28.21	28.16	28.72
	d	3.21	3.11	3.11	3.11	3.16	3.22	3.16	3.17	3.11
11	2θ	28.31	28.92	29.01	28.97	28.69	28.17	28.70	28.65	29.00
	d	3.15	3.09	3.08	3.08	3.11	3.17	3.11	3.12	3.08
12	2θ	28.78	30.52	30.67	30.47	29.00	28.67	29.00	28.92	30.56
	d	3.10	2.93	2.91	2.93	3.08	3.11	3.08	3.09	2.93
13	2θ	29.07	31.16	31.26	31.24	30.52	28.98	30.58	30.51	31.45
	d	3.07	2.87	2.86	2.86	2.93	3.08	2.92	2.93	2.84
14	2θ	30.65	31.39	34.23	34.22	31.22	30.52	31.25	31.19	34.18
	d	2.92	2.85	2.62	2.62	2.86	2.93	2.86	2.87	2.62
15	2θ	31.28	34.15	35.97	34.93	34.20	31.20	34.20	31.40	35.95
	d	2.86	2.63	2.50	2.57	2.62	2.87	2.62	2.85	2.50
16	2θ	31.51	34.94	37.11	35.89	34.93	31.41	34.95	34.13	37.10
	d	2.84	2.57	2.42	2.50	2.57	2.85	2.57	2.63	2.42
17	2θ	34.28	35.86	37.88	37.05	35.92	34.16	35.92	34.89	37.95
	d	2.62	2.50	2.38	2.43	2.50	2.62	2.50	2.57	2.37
18	2θ	35.01	37.04	39.41	37.92	37.06	34.93	37.08	35.88	39.41
	d	2.56	2.43	2.29	2.37	2.43	2.57	2.42	2.50	2.29
19	2θ	36.00	37.94	42.84	39.36	37.83	35.90	37.96	37.03	43.77
	d	2.50	2.37	2.11	2.29	2.38	2.50	2.37	2.43	2.07
20	2θ	37.13	39.33	43.77	42.78	38.02	37.06	39.38	37.91	47.79
	d	2.42	2.29	2.07	2.11	2.37	2.43	2.29	2.37	1.90
21	2θ	38.01	42.72	45.86	43.77	39.40	37.98	42.79	39.34	50.08
	d	2.37	2.12	1.98	2.07	2.29	2.37	2.11	2.29	1.82
22	2θ	39.42	43.70	47.85	44.72	42.79	39.39	43.85	42.77	51.88
	d	2.29	2.07	1.90	2.03	2.11	2.29	2.06	2.11	1.76
23	2θ	42.88	45.74	51.86	45.80	43.75	42.81	45.80	43.71	53.16
	d	2.11	1.98	1.76	1.98	2.07	2.11	1.98	2.07	1.72
24	2θ	43.83	47.77	52.78	47.85	45.78	43.76	47.79	45.74	54.33
	d	2.07	1.90	1.73	1.90	1.98	2.07	1.90	1.98	1.69
25	2θ	45.93	51.75	54.69	50.04	47.86	45.77	50.03	47.82	55.88
	d	1.98	1.77	1.68	1.82	1.90	1.98	1.82	1.90	1.65
26	2θ	47.88	53.07	56.79	51.78	51.85	47.77	51.26	51.75	56.78

	d	1.90	1.73	1.62	1.77	1.76	1.90	1.78	1.77	1.62
27	2θ	51.86	54.30	58.31	52.73	52.75	51.79	51.79	53.08	57.54
	d	1.76	1.69	1.58	1.74	1.74	1.77	1.77	1.73	1.60
28	2θ	53.19	56.76	59.15	54.65	53.11	53.09	52.97	54.27	63.66
	d	1.72	1.62	1.56	1.68	1.72	1.72	1.73	1.69	1.46
29	2θ	54.37	63.64	63.73	56.75	54.67	54.30	54.30	55.77	70.06
	d	1.69	1.46	1.46	1.62	1.68	1.69	1.69	1.65	1.34
30	2θ	56.78	70.08	65.14	58.28	56.74	56.70	54.68	56.73	72.15
	d	1.62	1.34	1.43	1.58	1.62	1.62	1.68	1.62	1.31
31	2θ	63.80	72.04	70.12	59.14	58.29	59.13	56.79	63.63	
	d	1.46	1.31	1.34	1.56	1.58	1.56	1.62	1.46	
32	2θ	70.12		72.35	63.67	59.16	63.65	58.29	70.05	
	d	1.34		1.31	1.46	1.56	1.46	1.58	1.34	
33	2θ	72.37		75.68	65.13	63.68	70.17	59.19	72.11	
	d	1.31		1.26	1.43	1.46	1.34	1.56	1.31	
34	2θ	75.94			70.09	65.05	72.29	63.69		
	d	1.25			1.34	1.43	1.31	1.46		
35	2θ				72.36	70.10		64.98		
	d				1.30	1.34		1.44		
36	2θ					72.28		69.98		
	d					1.31		1.34		
37	2θ							72.21		
	d							1.31		

(Note: #1 is the sample of Aba Prefecture Tibetan Medicine Hospital; #2 is the sample of Gan’na Prefecture Tibetan Medicine Hospital; #3, #4 and #5 are the samples of the Company of Tibetan Medicine of Tibetan Traditional Medical College; #6, #7 and #8 are the samples of Qinghai Province Tibetan Medicine Hospital; #9 is the sample of the Company of Tibetan Medicine of Tibetan Autonomous Region).
